# Evolutionary rescue: linking theory for conservation and medicine

**DOI:** 10.1111/eva.12221

**Published:** 2014-10-15

**Authors:** Helen K Alexander, Guillaume Martin, Oliver Y Martin, Sebastian Bonhoeffer

**Affiliations:** 1 Institute for Integrative Biology, D-USYS, ETH ZürichZürich, Switzerland; 2 Institut des Sciences de l'Evolution, UMR 5554, Université Montpellier 2 – CNRS – IRDMontpellier Cedex, France

**Keywords:** drug resistance, eco-evolutionary feedback, environmental change, experimental evolution, infectious disease, mathematical model, spatiotemporal heterogeneity, standing genetic variation

## Abstract

Evolutionary responses that rescue populations from extinction when drastic environmental changes occur can be friend or foe. The field of conservation biology is concerned with the survival of species in deteriorating global habitats. In medicine, in contrast, infected patients are treated with chemotherapeutic interventions, but drug resistance can compromise eradication of pathogens. These contrasting biological systems and goals have created two quite separate research communities, despite addressing the same central question of whether populations will decline to extinction or be rescued through evolution. We argue that closer integration of the two fields, especially of theoretical understanding, would yield new insights and accelerate progress on these applied problems. Here, we overview and link mathematical modelling approaches in these fields, suggest specific areas with potential for fruitful exchange, and discuss common ideas and issues for empirical testing and prediction.

## Introduction

Anthropogenic effects – including climate change, expanding land use and pollution (Millennium Ecosystem Assessment Board 2005) – are rapidly altering species' habitats. Loss of species unable to survive these changes could affect ecosystem services both directly through their individual contributions and indirectly via their role in ecosystem functioning (Chapin et al. 2000). In particular, biodiversity is thought to have a stabilizing effect on ecosystem services (Chapin et al. 2000; Hooper et al. 2005), evident on both between-species and within-species levels, such that even local extinctions of populations could threaten services such as fisheries (Schindler et al. 2010). Understanding how and why populations either persist or decline towards extinction is crucial to conservation efforts.

A superficially different, but likewise pressing, challenge arises in medicine. In chemotherapy, the desired outcome is eradication of a population of pathogens or cancerous cells. However, evolutionary responses frequently lead to drug resistance, compromising treatment of infectious diseases (Goldberg et al. 2012) and cancer (Bock and Lengauer 2012). Infections with antibiotic-resistant bacteria, for instance, are associated with higher morbidity and economic costs than those with antibiotic-sensitive strains (World Health Organization 2014).

A common thread links these problems: evolutionary adaptation occurring on the same timescale as demographic dynamics determines whether populations survive severe environmental change. In this scenario, prevention of extinction through genetic adaptation has been dubbed ‘evolutionary rescue’ (Gonzalez et al. 2013) (definitions are collected in Box 1). Although the term originates in conservation biology, it is equally applicable to scenarios where the goal of intervention is eradication. The latter case, the evolution of resistance to pesticides or drugs, is an important problem in agriculture as well as human health (REX Consortium 2007, 2010, 2013; Hendry et al. 2011), but to maintain a manageable scope, we focus on viral and bacterial pathogens and cancer, on the individual patient scale (although there are strong parallels to be found on the epidemiological scale). While restricting our focus within this field, we will make a novel connection between drug resistance and rescue in the conservation context.

**Box 1: Glossary**

*Evolutionary rescue*: genetic adaptation within a population facing environmental stress, allowing demographic recovery where otherwise extinction would occur. In the context of chemotherapeutic treatment of disease, this phenomenon is also called ‘emergence’ of drug resistance.

*Phenotypic plasticity*: the ability of an individual, with a given genotype, to express a range of phenotypes, particularly in response to different environments.

*Resistant/viable*: a genetic variant having positive net growth rate under a given environmental condition in which the wild type (sensitive ancestor) decays. Note that this definition puts a condition on absolute fitness, not only higher relative fitness than the ancestor. This term is applied here in the context of any stressful environment.

*Standing genetic variation* (SGV): genetic variation existing within a population prior to an environmental change. ‘SGV’ tends to be used in conservation and population genetics, whereas drug-resistant variants are typically said to ‘pre-exist’ in medical contexts.

De novo *variation*: genetic variation generated by mutation (or other processes such as recombination) after the onset of an environmental change, as opposed to standing or pre-existing prior to this change. In medical contexts, such mutants are sometimes said to be ‘induced’ by drug treatment.

*Reaction norm*: the phenotype or trait value of an individual or genotype, as a function of an environmental variable. The resulting phenotype reflects environmental effects and plasticity in the organism's response.

*Pharmacokinetics*: temporal pattern of drug concentration within a patient's body, due to dosing patterns and physiological processes such as uptake and breakdown of the drug.

*Pharmacodynamics*: effects of drug(s) on a targeted pathogen population. The effect, in particular pathogen replication or death rate, as a function of drug concentration is called a ‘dose–response curve’. This can be seen as a particular kind of reaction norm, where the response variable is usually a demographic parameter.

*Malthusian fitness*: expected instantaneous net exponential growth rate of a genotype; in other words, the rate of change of the log size of the subpopulation carrying a given genotype. In a simple linear birth–death process, this is birth rate minus death rate.

*Cost of resistance*: selection coefficient, that is reduction in fitness, of a resistant/viable mutant compared to the wild type (sensitive ancestor) in permissive conditions (the ancestral environment). In a continuous-time model, this is the mutant's absolute reduction in Malthusian growth rate, while in a generation-based model, it is measured by proportional reduction in surviving offspring.

Conservation biology focuses on small populations, often of obligately sexual, multiploid organisms with long generation times. Asexual unicellular organisms performing key ecosystem functions, such as marine phytoplankton performing calcification (Lohbeck et al. 2012), also face pressure to adapt to changing conditions but not a comparable threat of extinction. Small populations face particular problems with genetic variance and individual fitness, including inbreeding in sexual populations (Willi et al. 2006; Bijlsma and Loeschke 2012). *De novo* mutations are rare (roughly averaging on the order 10^−8^ per base pair per generation for plants and animals, although with variation among taxa; Lynch 2010), but recombination and segregation can generate genetic diversity by shuffling existing alleles to create new haplotypes. Meanwhile, multiploidy implies that expression of alleles is affected by dominance. Many organisms of conservation concern have complex life histories and considerable scope for adaptive plasticity and dispersal. Global environmental change, even when rapid relative to historical levels, remains slow on the timescale of human observation and challenging to predict.

Medical treatments, in contrast, deal with large populations of organisms that replicate rapidly and primarily asexually. Mutation rates are high in many populations of interest, notably cancer cells, which are characterized by genetic instability (Hanahan and Weinberg 2000), and RNA viruses (Sanjuan et al. 2010). To take human immunodeficiency virus (HIV) for example, a mutation rate of 3 × 10^−5^ per base pair per replication (Mansky and Temin 1995), combined with estimates of population size and generation time, imply that any single-point mutation – often sufficient for single-drug resistance – is expected to arise in an untreated patient many times daily (Coffin 1995). Recurrent *de novo* mutation is thus an important source of genetic diversity, although many bacteria engage in horizontal gene transfer (both intra- and interspecific), and certain viruses, within multiply-infected cells, can recombine, reassort or complement one another (akin to dominance in polyploids). Plasticity and dispersal, although relevant to disease (Levin and Rozen 2006), are considered less central than genetic change. Environmental change is directly controlled, and molecular mechanisms of drug action and resistance are often known.

We briefly note that agricultural applications provide in many features a middle ground of biological systems. Akin to conservation, the target organisms are behaviourally plastic, actively dispersing, multiploid eukaryotes with sexual reproduction, including both multicellular and unicellular species. Akin to medical applications, their demography approaches that of microbial human pathogens, with relatively large populations and short generation times. Furthermore, a targeted chemotherapy is applied with the goal of eradication, and the genetic basis of resistance is often clear. Linking agronomy to the study of evolutionary rescue thus has considerable potential, although we do not delve into the subject in detail here. Connections between agricultural and medical models have been discussed elsewhere (REX Consortium 2007, 2010, 2013; van den Bosch and Gilligan 2008).

**Figure 1:**
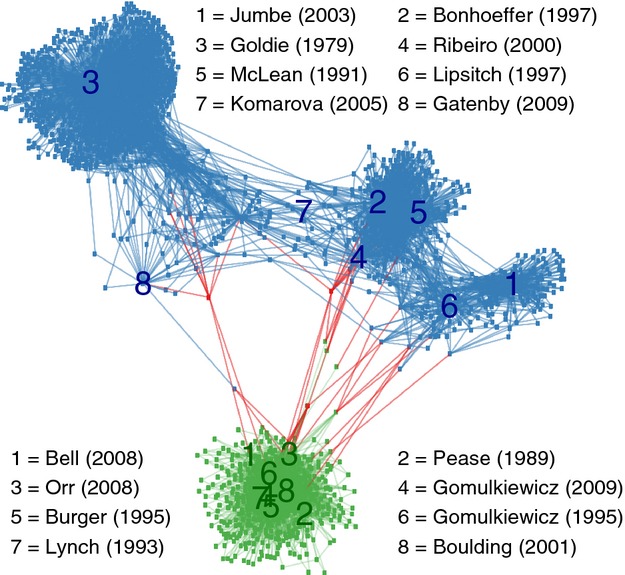
Citation network. We first selected eight highly cited and influential theoretical papers on each side – conservation (Pease et al. 1989; Lynch and Lande 1993; Bürger and Lynch 1995; Gomulkiewicz and Holt 1995; Boulding and Hay 2001; Bell and Collins 2008; Orr and Unckless 2008; Gomulkiewicz and Houle 2009) and drug resistance (Goldie and Coldman 1979; McLean et al. 1991; Lipsitch and Levin 1997; Bonhoeffer et al. 1997; Ribeiro and Bonhoeffer 2000; Jumbe et al. 2003; Komarova and Wodarz 2005; Gatenby et al. 2009) – reflecting a diversity of subtopics, approaches and authors. These 16 key papers are labelled at their locations in the citation network. The network also includes as nodes those papers that cite at least one of these 16 starting papers, according to Web of Knowledge indexing as of September 2013. Two nodes are connected by an edge if one cites the other. Any nodes with a single link are removed, firstly for visual clarity and secondly to avoid including studies only peripherally connected to the topic. A node and its edges are coloured blue if the paper is included due to citation of key paper(s) on the drug resistance side only; green if on the conservation side only; or red if citing at least one key paper on each side. The graph is arranged by applying the Fruchterman–Reingold algorithm available in the R package igraph (Csardi and Nepusz 2006). The network is thus an illustration of connections, or lack thereof, among primarily theoretical literature. Subfields within the drug resistance side (cancer, viruses, bacteria) are substantially better connected with one another than the drug resistance and conservation sides are connected to each other.

With such contrasting systems and goals, it is perhaps unsurprising that the fields of conservation and medicine have largely led separate existences. Mathematical modelling has been a key tool for developing understanding on both sides. However, despite awareness that resistance to chemotherapy is an example of evolutionary rescue (Bell and Collins 2008; Gonzalez et al. 2013; Lindsey et al. 2013; Martin et al. 2013; Ramsayer et al. 2013; Carlson et al. 2014; Orr and Unckless 2014; Uecker et al. 2014), mathematical approaches in conservation versus medical contexts are *de facto* rather disconnected. In the theoretical literature, cross-citations remain rare (informally illustrated in Fig. 1) despite conceptual similarities and common findings. Furthermore, we are not aware of any review integrating models from both sides. Nonetheless, several fundamental questions are relevant in both applied fields and could be better understood through cross-community discussion. In this article, we aim to raise awareness of this potential by reviewing modelling approaches in each field, delineating links between them, and suggesting specific areas that could benefit from transferring ideas and techniques.

## A common conceptual basis

Consider the scenario where a population faces an environmental change sufficiently severe that the population will decline and face extinction unless it responds. There are several, not mutually exclusive possibilities to promote survival, including dispersal to more favourable environments or plastic responses (Levin and Rozen 2006; Barrett and Hendry 2012), as well as adaptation through genetic change. These mechanisms can interact in determining the fate of a population (Chevin et al. 2010; Reed et al. 2011; Schiffers et al. 2013; Merilä and Hendry 2014). Here, however, we focus on the contribution of evolution to local adaptation.

The importance of evolution in rescuing populations is unequivocal in medical settings, but remains unclear in conservation settings (Barrett and Hendry 2012). Evolution is argued to be relevant for some vertebrates in the wild (Vander Wal et al. 2013), and indeed, there are examples of rapid evolution in natural populations (Ferrière et al. 2004; Gonzalez et al. 2013). However, practical challenges in collecting and interpreting data (Gomulkiewicz and Shaw 2013), including the difficulty of ascertaining whether phenotypic change has a genetic basis (Merilä and Hendry 2014), have resulted in few clear-cut empirical examples. Nonetheless, long-term survival of populations facing severe environmental change is expected to require evolution, due to limits of plasticity and barriers to dispersal (Frankham and Kingsolver 2004; Visser 2008; Chevin et al. 2010; Barrett and Hendry 2012; Schiffers et al. 2013). Furthermore, a growing body of theoretical work deals with the possibility of evolutionary rescue.

Declining populations face a ‘race’ between adaptation and extinction (Gomulkiewicz and Holt 1995; Orr and Unckless 2008). Moreover, in comparison to a population remaining stable at the initial size, fewer new genetic variants are generated and beneficial mutations with a given selection coefficient are more likely to be lost in a declining population (Otto and Whitlock 1997). Thus, unlike in classical population genetic approaches, the possible extent of evolution is limited by demography (Gomulkiewicz and Houle 2009). Conservation biology and medicine both aim to understand these dynamical processes, ultimately to predict whether rescue will occur and intervene effectively. A mutual goal is to identify factors affecting the outcome. Although sometimes couched in different terms, essentially equivalent parameters are highlighted, including severity of environmental change (e.g. drug dosage or rate of temperature increase), population size, and genetic variance or mutation rate.

A natural follow-up is to identify the source of rescue. As the predominant ancestral genetic variant (wild type) has low fitness in the new environment, rescue requires outgrowth of some ‘resistant/viable’ variant(s) having sufficiently high fitness. (We will use the term ‘resistant/viable’ throughout the article as shorthand for a genotype having positive expected growth rate in the novel environment.) One can then ask whether resistant/viable lineages rescuing the population tend to pre-exist, that is, come from the standing genetic variation (SGV) before the environment changed, or arise *de novo* after the environmental change. This question has been raised and has applied implications in both conservation (Orr and Unckless 2008; Barrett and Hendry 2012) and chemotherapy (Bonhoeffer and Nowak 1997; Lipsitch and Levin 1997; Ribeiro and Bonhoeffer 2000; Komarova and Wodarz 2005; Read et al. 2011).

Although the mathematical study of evolutionary adaptation has a rich history, ecological and evolutionary timescales were traditionally separated (Ferrière et al. 2004; Bell 2013), resulting in a relative dearth of models incorporating simultaneous change and bidirectional feedback between demography and genetics. Theoreticians motivated by applications in conservation versus medicine have often taken different approaches to address evolutionary rescue, reflecting biological differences in systems of interest, but also historical developments. Within each field, substantial differences among organisms have not prevented development of generic models on the conservation side, or fruitful exchange of questions and techniques on the more organism-specific medical side (e.g. Goldie and Coldman 1979; Komarova and Wodarz 2005). We argue that exchange within the theoretical community could usefully be extended across fields. Below, we briefly review the most widespread and influential modelling approaches on each side, then illustrate through detailed comparison of two models how connecting divergent fields is promising.

## Mathematical modelling in two fields

An ‘evolutionary rescue’ model has three essential ingredients: (i) it incorporates a (severe) change in the extrinsic environment; (ii) it describes the temporal dynamics of both genetics and demography, including how they affect each other; and (iii) it addresses extinction risk. Different schools of modelling have incorporated these ingredients in different ways.

### Conservation

Models motivated by conservation scenarios often extend population genetics approaches, fundamentally concerned with allele frequencies, to add demographic change determined by absolute fitness, as opposed to extrinsically imposing a total population size. Fitness is usually taken to be density-independent in analytical approaches (but see Uecker et al. 2014), an assumption sometimes relaxed in simulations (Bürger and Lynch 1995; Boulding and Hay 2001; Orr and Unckless 2008). Population dynamics are formulated either in discrete, nonoverlapping generations or in continuous time. Models can be subdivided according to the supposed genetic basis of adaptation: continuous (quantitative) or discrete.

Quantitative genetic models suppose that many cosegregating alleles contribute to a continuous-valued trait. These models typically describe sexual organisms with many biallelic loci segregating independently (free recombination), but can also apply to asexual organisms with a high mutation rate (hence many alleles at one ‘locus’). Analytical approaches usually assume a Gaussian distribution of trait values in the population, described by its mean and phenotypic variance (Lynch and Lande 1993; Gomulkiewicz and Holt 1995), while simulations determine individuals’ trait values from a finite number of loci contributing specified effect sizes (Bürger and Lynch 1995; Boulding and Hay 2001). A portion of phenotypic variance is due to additive genetic variance, on which selection can act. The dynamics of genetic variance, particularly its connection to demography, have been treated in various ways: assumed constant (Lynch and Lande 1993; Gomulkiewicz and Holt 1995); modelled by mutation-selection-drift equilibrium (Bürger and Lynch 1995); or directly determined from genetic composition in simulated populations (Bürger and Lynch 1995). Meanwhile, fitness is taken as a function of trait value, decreasing with distance from an optimum. Environmental change has been treated in two ways: continual or abrupt. The former is modelled by shifting the optimal trait value at a constant rate (Lynch and Lande 1993). Response to selection allows the population mean trait value to track the moving optimum, asymptotically establishing a constant lag distance. However, if environmental change exceeds a critical rate (determined by factors including available genetic variance and width of the fitness function), the population's mean fitness at this lag is negative and it faces deterministic extinction. Demographic, genetic and environmental stochasticity can further contribute synergistically to population demise even when the rate of environmental change is below the deterministic extinction threshold (Bürger and Lynch 1995). In the treatment of abrupt environmental change, populations adapt towards a novel but fixed optimum (Gomulkiewicz and Holt 1995; Boulding and Hay 2001). Critical population size has been used in deterministic analytical treatments to set a heuristic threshold criterion for extinction vulnerability (Gomulkiewicz and Holt 1995), while by simulation of a finite population, extinction can be observed directly (Boulding and Hay 2001). Analytical approaches have been extended to multivariate traits, characterized by a matrix of genetic covariances, with environmental change (continual or abrupt) implemented as a shift of optimum in multidimensional trait space (Gomulkiewicz and Houle 2009). For further details on quantitative genetic models of adaptation (not exclusive to rescue), including recent extensions to the basic framework, we refer the reader to a recent review (Kopp and Matuszewski 2014).

Discrete genetic models classify population members into a small set of genotypes (determined by one or few loci) with specified fitness values. These models typically consider a single, abrupt environmental change. One deterministic approach (Gomulkiewicz and Holt 1995) considers a diploid genetic system where one biallelic locus determines fitness. The well-adapted allele in the novel environment starts at nonzero frequency, so selection acts on SGV, resulting in allele frequency change described by standard population genetics results. As in the aforementioned models, demographic change is determined by population mean fitness and critical population size is used to indicate extinction risk. Other discrete models have treated population dynamics stochastically, focusing on the probability of rescue from SGV and/or *de novo* mutations (Bell and Collins 2008; Orr and Unckless 2008; Martin et al. 2013; Uecker et al. 2014), although demographic trajectories through time can also be described (Orr and Unckless 2014). SGV is typically captured by a mutation-selection(-drift) equilibrium, while occurrence of *de novo* mutations is proportional to wild-type population size, which declines at a fixed rate. The spread of beneficial alleles is then modelled as a branching process.

Besides continuous versus discrete genetics, these two classes differ on the level at which adaptation is modelled (Orr 2005). The first considers a continuous phenotype under stabilizing selection, and sets mutation rates and effects on phenotype, the population's distance from the optimum, and a phenotype-to-fitness mapping. The second approach directly sets mutational rates and effects on fitness, or components thereof (demographic parameters under directional selection), in each environment. This association is historical but not necessary: one could model a continuum of alleles directly affecting fitness, or a discrete phenotypic landscape.

We finally mention another school of modelling more closely linked to the conservation side, broadly known as evolutionary ecology. Despite the common interest in linking population genetics and dynamics, the techniques developed here have so far hardly been used to address the evolutionary rescue scenario, that is, incorporate the possibility of extinction due to extrinsic environmental change. Evolutionary ecology recognizes that the ‘environment’ is not only an extrinsic factor, but also shaped by ecological feedbacks (Day 2005; Ferrière and Legendre 2013). In particular, the fitness landscape is not fixed, but can depend on total population density and/or genotype frequencies (Day 2005; Waxman and Gavrilets 2005). This concept has been incorporated into various, typically deterministic, mathematical frameworks rooted in population genetics and game theory (Day 2005). Particularly prominent among these is adaptive dynamics (AD), which has made great strides in gaining analytical insights, although under rather strong assumptions (rare mutations of small effect; Waxman and Gavrilets 2005). The extension of AD to evolutionary rescue has recently been discussed (Ferrière and Legendre 2013) and implemented in one model (Osmond and de Mazancourt 2013). However, the separation of ecological and evolutionary timescales inherent to AD (Day 2005) necessitates a heuristic consideration of extinction on the fast demographic timescale. Another approach to evolutionary ecology, which does not separate timescales, has been developed in a series of papers coupling quantitative or discrete population genetic approaches, including Price equation formulations, to demographic models that generally include ecological feedbacks (Day and Proulx 2004; Day and Gandon 2006, 2007, 2012; Gandon and Day 2009). Although the framework is quite general, these papers are particularly interesting in the context of connecting fields, as they have drawn examples from classical ‘compartmental’ epidemiological models (see next subsection), and the approach has also explicitly been applied to within-host pathogen evolution (Alizon 2009). Although not yet linked to evolutionary rescue, these and similar approaches could presumably be extended to consider extrinsic environmental change and extinction risk in the same way as the aforementioned population genetics-based models.

### Drug resistance

Most models dealing with drug resistance can be described as ‘population dynamics’ approaches, fundamentally concerned with demography, and extended to consider genetic heterogeneity by subdividing the relevant population into drug-sensitive and drug-resistant variants with distinct demographic parameters. These models typically work in continuous time and nearly always consider an abrupt environmental change (absence to presence of drugs).

One popular approach starts from a ‘compartmental’ description of flow in and out of (possibly several) populations, often taking into account the organism's life cycle and including density-dependent processes. The mathematical formulation is a system of ordinary differential equations (ODEs). With close connections to epidemiological-scale modelling (Anderson and May 1991), this approach is most common for pathogens within an infected host, such as viruses (McLean et al. 1991; Bonhoeffer et al. 1997; Nowak and May 2000) or bacteria (Lipsitch and Levin 1997; Jumbe et al. 2003), but also used for cancer (Michor et al. 2005). Stochasticity is typically considered by simulating the demographic events described by the ODEs (e.g. Ribeiro and Bonhoeffer 2000), using variations of the Gillespie algorithm (Gillespie 1977). Rarely, approximating the dynamics of the mutant population by simpler stochastic processes has allowed analytical results (Alexander and Bonhoeffer 2012; Tomasetti 2012).

Another approach describes population dynamics directly by stochastic processes. Specifically, most models use branching processes, assuming density-independent growth (but see Sorace and Komarova 2012). This body of work is rooted in early laboratory studies seeking to understand the process of bacterial mutation (Luria and Delbrück 1943; Lea and Coulson 1949). The ‘Luria–Delbrück’ distribution describing the number of mutants appearing in a growing population before screening is widely applied in estimating mutation rates from fluctuation assays (see Empirical approaches), including long-standing consideration of drug resistance mutations (David 1970). Mutations are often assumed to be cost free, although this assumption can be relaxed (Zheng 1999). In parallel, the mathematical derivation has been adopted and extended for purely theoretical investigations into the emergence of drug resistance. Applications have primarily been to cancer, beginning with the pioneering work of Goldie and Coldman (1979, Coldman and Goldie 1983, 1986) and remaining an active topic (Komarova and Wodarz 2005; Iwasa et al. 2006); see Foo and Michor (2014) for a recent review. Less commonly, the approach has been applied to viral infections (Haeno and Iwasa 2007) and bacterial infections (Colijn et al. 2011). These models have retained a focus on deriving the number of mutants in a population growing to a given size before treatment, thus fundamentally dealing with SGV, although occasionally also considering *de novo* production of mutants during treatment (Komarova and Wodarz 2005; Colijn et al. 2011).

The two aforementioned approaches differ in their typical treatment of stochasticity, inclusion of density dependence, and assumption of demographics prior to the onset of drug treatment (equilibrium versus exponential growth; cost of resistance). Nonetheless, by taking a discrete set of strains and directly specifying fitness or demographic parameters, both categories of drug resistance models align closely with discrete population genetics models on the conservation side. We make this connection more concrete in the next section.

We briefly remark that models used to investigate emergence of resistance in agricultural settings share a number of similarities with those used in medical settings. In particular, resistance is rarely considered as a quantitative trait (REX Consortium 2010). The parallels between models of fungicide and of antibiotic resistance have been emphasized by van den Bosch and Gilligan (2008). Nonetheless, the medical and agronomic modelling communities are rather separate (REX Consortium 2007), and several biological and technical features have been considered to significantly different extents for different organisms (REX Consortium 2010).

## Linking models

The modelling literature reviewed above has developed rather independently in the two fields, building on previous contributions motivated by similar applications and rarely crossing over (Fig. 1). We propose, however, that rescue models on this level of simplification are more generic than the context in which they have thus far been placed and that the divide is more reflective of history and application than fundamental differences in biology or mathematical structure.

We illustrate this point by examining parallels between two recent rescue models (Alexander and Bonhoeffer 2012; Martin et al. 2013). Despite being rare examples touching on the interface between fields, each clearly shows a closer association to a different body of literature. These studies are amenable to comparison because they share a number of similar structural features: both deal with stochastic population dynamics in a scenario of abrupt environmental change and discrete adaptive steps. However, their contrasting approaches make it nontrivial to elucidate what turn out to be equivalent results.

The model by Martin et al. (2013) exemplifies the ‘discrete genetics’ approach within the conservation-based school of modelling. Although it is applied to experimental populations of microbes, its theoretical references are drawn primarily from the conservation-motivated literature. It tracks a single population with generic life history rather than specifying particular biological interactions, and describes density-independent dynamics (mathematically, a branching process). A single mutational step from the wild type is sufficient to confer resistance/viability in the novel environment, but this step can land on an arbitrary set of distinct variants, characterized by a distribution of growth rate, reproductive variance and selective disadvantage in the original environment.

The model by Alexander and Bonhoeffer (2012) is based on the ‘compartmental’ approach to drug resistance models, generalizing a widely used within-host viral dynamics model (Nowak and May 2000). The system can be described deterministically by ODEs or translated to stochastic events, the latter being our focus here. The replication cycle involves two steps: free virus infects target cells and infected cells in turn produce free virus. There are two specified strains, drug-sensitive and drug-resistant, and mutation to the resistant strain can occur at either step of the cycle. Fitness is a composite of replication cycle parameters, and fitness differences between strains (due to drug action and cost of resistance) can arise through various traits. Furthermore, between-strain competition for target cells, and thus density dependence, emerges in this model. Although the focus on stochasticity is unusual for compartmental-based drug resistance models, it typifies the model formulation in this class.

Both studies address the probability that rescue occurs from SGV existing in the original, ‘permissive’ (no drug) environment, or from *de novo* mutations occurring in the new, ‘stressful’ (drug) environment. Each study derives analytical approximations of these probabilities from simplified stochastic processes. It turns out that both sets of results fit into a general expression to which each model makes a different simplification (Box 2). This encompassing framework leads to several insights.

**Box 2: A common mathematical framework**

**Figure 2:**
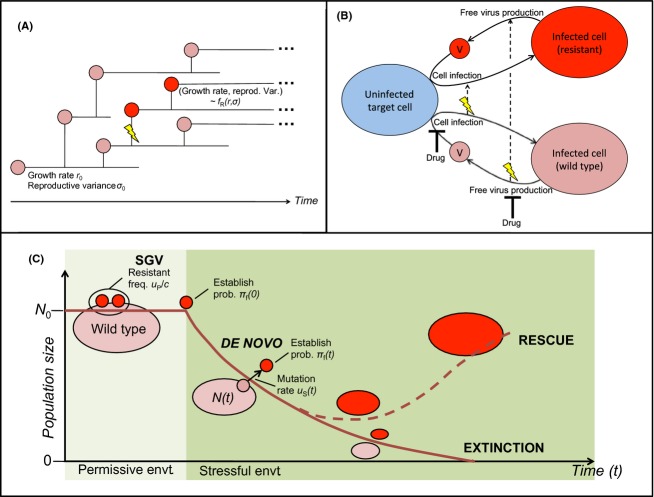
Models of rescue from SGV and *de novo* mutations. (A) A branching process model of population growth, whose dynamics can be described by a diffusion approximation (Martin et al. 2013). The wild type (pink) has mean growth rate *r*_0_ and reproductive variance *σ*_0_. At a reproduction event, mutation (lightning bolt) to a resistant variant (red) can occur. This variant has reproduction parameters drawn from a specified distribution *f*_*R*_. (B) A viral dynamics model (Alexander and Bonhoeffer 2012). Free virus (V) infects target cells; infected cells in turn produce free virus. Two strains, drug-sensitive (wild type) and drug-resistant, are characterized by their distinct rates of replication cycle events. Mutation (lightning bolt), for clarity shown only from the wild type, can occur upon either cell infection or free virus production. Under treatment, a drug can block the sensitive strain at either of these replication steps (inhibition arrows). (C) Schematic of population size over time, leading to an outcome of either extinction or rescue. The size of ovals represents population size (pink, wild type; red, resistant), while circles indicate individuals within the population. Resistant variants leading to rescue can arise from two sources. (i) Standing genetic variation (SGV): Resistant individuals are maintained at mutation-selection balance under permissive conditions. After the switch to stressful conditions, a resistant individual is at a selective advantage and succeeds in establishing a lineage with probability *π*_*f*_(0). (ii) *De novo* production: Under stressful conditions, the wild-type population (size *N*(*t*)) declines, but residual replication leads to ongoing production of resistant mutants at per capita rate *u*_*S*_(*t*). A resistant individual arising at time *t* has probability of establishment *π*_*f*_(*t*).

The first model under consideration (Martin et al. 2013) uses a diffusion approximation to model density-independent population dynamics in discrete or continuous time (Fig. 2A). An arbitrary set of possible variants (genotypes) are each characterized by their mean growth rate (*r*) and reproductive variance (*σ*) under stressful conditions, and cost relative to the wild type under permissive conditions (*c*), which may be related to *r* and *σ*. Upon replication, the wild type mutates with given probability to a resistant variant with parameters drawn from the distribution *f*_*R*_(*r*, *σ*).

**Figure 3:**
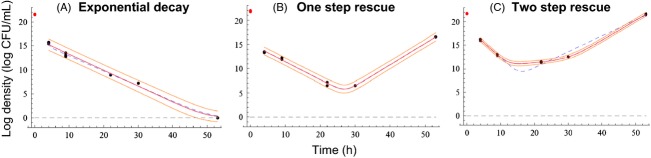
Alternative dynamics of *Pseudomonas fluorescens* populations facing streptomycin stress. The plots present three illustrative examples of demographic trajectories in bacterial populations exposed to an antibiotic, showing characteristic decline with possible rebound (rescue). Dots show measurements (data from Ramsayer et al. 2013) and lines indicate model fits (G. Martin, unpublished). Solid lines show the best-fitting model (red: best fit; orange: 95% confidence limits), while dashed blue lines show the alternative simpler model, where appropriate. (A) Density-independent, that is log linear, decline with extinction; (B) rescue involving one mutational step; (C) rescue involving two successive mutational steps. As the initial sharp drop in population size (red dot) indicates, real dynamics are slightly more complex than the simple model scenarios.

The second model (Alexander and Bonhoeffer 2012) considers a within-host viral infection (Fig. 2B). Two strains of virus, drug-sensitive and drug-resistant, are characterized by replication cycle parameters, namely the rates at which free virus infects cells, infected cells die, free virus is produced by infected cells, and free virus is cleared. Resistance bears a cost through any one of these parameters, while drug treatment blocks either cell infection or viral production of the sensitive strain with given efficacy. Mutation occurs with given probability upon either cell infection or viral production. Although the replication cycle is multistep, mathematically it suffices to track the population of infected cells, taking effective mutation rates and drug efficacies composed over the two-step cycle.

Figure 2C schematically illustrates the essential features of the rescue process common to both models**.** The population reaches mutation-selection balance in the permissive (drug-free) environment, followed by a switch to stressful (drug treatment) conditions. Resistance arises via a single mutational step, and a ‘rescue mutant’ is a resistant individual whose lineage escapes stochastic extinction, that is, establishes in the stressful environment. The general results presented below encompass the results of both models in this scenario.

*Rescue by standing genetic variation*: Provided the probability of establishment per individual is small, the probability of rescue by SGV is approximately:

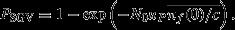

Three parameters determine the mutation-selection balance under permissive conditions: total population size, *N*_0_; per capita rate of mutation from wild type to resistance per unit time, *u*_*P*_; and cost of resistance, *c*. If mutation occurs upon replication, *u*_*P*_ should be derived as the wild-type replication rate times probability of mutation per replication, as in the viral dynamics model (Alexander and Bonhoeffer 2012). In expectation (which suffices for the approximation; Martin et al. 2013), *N*_0_ *u*_*P*_/*c* resistant individuals pre-exist in the permissive environment. Then, *π*_*f*_(0) is the probability of establishment of a single resistant individual in the stressful environment, if present at the onset of stress (time 0). Establishment probability can be approximated by the classical Feller diffusion result (Martin et al. 2013) or derived from a birth–death process accounting for dynamics of multiple populations (Alexander and Bonhoeffer 2012). The overbar denotes averaging over the distribution of mutational effects, *f*_*R*_(*r,σ*), if there are multiple resistant variants (Martin et al. 2013).

*Rescue by* de novo *mutation*: In the stressful environment, rescue mutants are produced *de novo* from the wild type according to a time-inhomogeneous Poisson process with the following rate at time *t*:

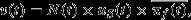

yielding the probability of rescue by *de novo* mutation:

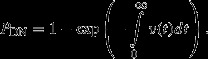

The rate of production of rescue mutation is determined by the product of: (i) the wild-type population size, whose decline can be approximated deterministically with the form *N*(*t*) = *N*_0_ exp(*R*(*t*)) in both models; (ii) per capita mutation rate per unit time in the stressful environment, *u*_*S*_(*t*); and (iii) probability of establishment, *π*_*f*_(*t*), again averaged over the distribution of mutational effects if necessary. In the simplest case, *R*(*t*) = *r*_0_*t* and the parameters *r*_0_, *u*_*S*_, and *π*_*f*_ can be assumed constant through time (Martin et al. 2013). However, ecological feedbacks, namely rebound of resources (target cells in the viral dynamics model) as the total population size declines, can introduce time dependencies (Alexander and Bonhoeffer 2012). For multistep life cycles, a correction term can be added to the expression for *P*_DN_ to account for a process neglected in the first approximation; see Alexander and Bonhoeffer (2012) for details.

Firstly, this explicitly shows that models with different starting points lead to the same key determinants of rescue: population size, mutation rate, and characteristics of resistant/viable variant(s) in both permissive and stressful environments. A comparison of equations even yields the stronger statement in this case that the parameter dependencies are the same, to the order of the given approximations. This link is actually more general than the two studies presented above: particular results derived by Bell and Collins (2008) and Orr and Unckless (2008) arise as special cases of the equations in Martin et al. (2013). Furthermore, the HIV model of Ribeiro and Bonhoeffer (2000), when restricted to a single locus, represents a special case of the model of Alexander and Bonhoeffer (2012), while the Hepatitis C Virus model of Rong et al. (2010) differs only in the dynamics of target cells.

Secondly, this suggests how simplifications made by each model could be extended using analytical tools from the other. For example, a single resistant variant (Alexander and Bonhoeffer 2012) can be reinterpreted as an ‘effective’ resistant variant averaged over a distribution of mutational effects (Martin et al. 2013). Mutation rates can differ under permissive versus stressful conditions (Martin et al. 2013) and total mutational influx can be derived from particular population dynamics (Alexander and Bonhoeffer 2012). The assumption of density-independent fitness (Martin et al. 2013) can be relaxed to incorporate ecological feedbacks through parameters that vary deterministically through time according to the population dynamics of dominant strain(s) (Alexander and Bonhoeffer 2012).

Nonetheless, this analysis also highlights where limitations of common modelling choices consistently arise (see also Areas for exchange and future work). Both models assume simplistic population history generating SGV and step changes in environment. We expect that the latter could be extended using the analytical framework of Uecker and Hermisson (2011), which derives the fixation probability of beneficial alleles for arbitrary time-varying population size and selection coefficients (see their Appendix for the rescue scenario). Perhaps more crucially, resistance/viability in both models is achieved by single mutations. However, both empirical (e.g. Marcusson et al. 2009) and theoretical (G. Martin, unpublished) results suggest that multistep rescue might be important, especially when the environmental change is severe.

Making links between further categories of models is less straightforward, due to fundamental differences in model structure: the assumption of quantitative genetics and a trait-based landscape, versus discrete genetics and direct specification of fitness. The different nature of outputs from deterministic versus stochastic models also prevents a direct comparison of equations. However, similar qualitative features – including large population size, high standing genetic variance and/or mutation rate, and less severe environmental change – have been noted to promote rescue in different mathematical frameworks (Gomulkiewicz and Holt 1995; Bell and Collins 2008; Osmond and de Mazancourt 2013), although more biologically complex models, for instance incorporating ecological feedbacks, can sometimes alter predictions (see the discussion in Ferrière and Legendre 2013 and ‘Optimal selection strength’ below). Several of these features have also been tested experimentally (see Empirical approaches).

## Forecasting from models

All evolutionary models face a challenge in prediction: they rely on the demographic parameters and rate of appearance of yet-to-appear genotypes. In the rescue setting, these parameters are required both before and after the environmental change, to predict the contributions from SGV and *de novo* mutations, respectively. Put generally, the joint distribution of mutational rates and effects across genotypes and environments is required (Martin et al. 2013), expanding to many environments in models with spatial structure or continual temporal change. Context dependence of mutational fitness effects is notoriously pervasive and complex (Fry and Heinsohn 2002; Agrawal and Whitlock 2010), making it difficult to extrapolate from a few measurements.

One option to tackle model parameterization is direct measurement of the entire required distribution of mutational rates and effects, a tedious but increasingly feasible possibility for species that can be studied in the laboratory at high throughput (see Empirical approaches). Alternatively, one can try to predict the distribution using fewer measurements. Prediction from a mechanistic model (e.g. a metabolic model, Papp et al. 2011) is occasionally an option, but with limited extensibility to nonstandard conditions. Alternatively, one can use a model relating fitness effects across environments, for example phenotype-fitness landscape models, which provide the required prediction so long as different environments simply reflect shifts in optima with limited change in the shape of the landscape (Martin and Lenormand 2006). The category of quantitative genetic rescue models described above happens to rely on such an underlying landscape. Measurements required to parameterize such models are reduced to mutational rates and effects in one baseline environment and the decay rate of the wild type in each environment. Nevertheless, whether the prediction is accurate remains an open question for empirical testing.

## Empirical approaches

Our focus has been on the insights to be gained through theoretical studies. Nonetheless, empirical work is crucial to test whether model assumptions are valid, predictions hold up, or extensions are necessary, as well as to parameterize models for forecasting.

Experimental evolution is a powerful tool for testing models in a controlled setting, delineating parameter effects more clearly than in field or clinical settings. Despite abundant studies documenting trajectories of evolutionary adaptation and its underlying mechanisms, fewer experiments explicitly focus on evolutionary rescue, in particular where the wild type is expected to decline in the novel environment and population extinction is allowed. The shared set of laboratory model systems (usually using microbes) and concerns for experimental design again yield common ground between conservation and medical fields.

Rescue experiments can provide various information, including the proportion of replicates surviving stress, yielding estimates of rescue probability (Bell and Gonzalez 2009, 2011; Lachapelle and Bell 2012; Lindsey et al. 2013); population dynamics over time, allowing model fitting and parameter estimation (Martin et al. 2013; Ramsayer et al. 2013); and/or genetic characterization (Lindsey et al. 2013), hinting at underlying mechanisms. Recent studies have demonstrated key determinants of rescue (see also Carlson et al. 2014 for a review): inoculum size (Bell and Gonzalez 2009; Samani and Bell 2010; Ramsayer et al. 2013), initial diversity (Lachapelle and Bell 2012; Ramsayer et al. 2013), evolutionary history in sublethal stress (Samani and Bell 2010; Lachapelle and Bell 2012; Gonzalez and Bell 2013; Lindsey et al. 2013), rate or extent of environmental change (Perron et al. 2008; Collins and de Meaux 2009; Toprak et al. 2012; Lindsey et al. 2013), sexual versus asexual reproduction (Lachapelle and Bell 2012), and population connectivity (Perron et al. 2007, 2008; Bell and Gonzalez 2011).

The following methodologies provide examples of insights gained from analysis of experimental data. We focus on pointing out which data are crucial to collect in order to compare experimental results to theory and to gain general insights into the rescue process.

### Fluctuation assay

Several populations are grown in permissive medium, then plated independently on selective medium. The resulting distribution of colony counts per plate allows estimating the rate and cost (in permissive medium) of mutations conferring growth in the selective medium (Luria and Delbrück 1943; Hamon and Ycart 2012). This allows parameterizing and testing models of adaptation from SGV.

### Genetic sequencing and strain construction

Sequencing resistant/viable lines (recovered from fluctuation assays, long-term evolution experiments, or clinical samples) allows identification of the genetic basis of adaptation, including whether sites of genetic change are consistent or diverse, and whether single or several mutations are involved (Toprak et al. 2012; Lindsey et al. 2013). Where possible, constructing and testing strains containing only particular mutations on a wild-type background can confirm these mutations' role in adaptation (e.g. Marcusson et al. 2009), or alternatively suggest that other, unidentified mutations also contributed.

### Demographic parameters across environments

Net growth rate of an experimental population can be estimated from measurements of population size over time, obtained from standard assays (e.g. optical density in a cell culture well). Novel techniques (e.g. fluorescent stains that differentially mark live and dead cells; Berney et al. 2007) also allow the separate estimation of replication and death rates, which according to models can play distinct roles in determining mutational influx and rescue dynamics. Furthermore, these measurements can be made for populations exposed to various environments, generating a ‘reaction norm’ or ‘dose-response curve’ as a function of a continuous environmental parameter. In a drug resistance context, this parameter would be drug concentration, with measurements yielding pharmacodynamic functions (Regoes et al. 2004).

### Quantifying stress

In both clinical studies and experimental evolution, ‘stress’ is frequently quantified as some physical measure of the environmental parameter (e.g. drug concentration or temperature) and ‘rate of environmental change’ by the rate of change in this physical measure. However, the effect of these stresses on rescue depends on how they affect (i) fitness or demographic parameters (birth and death rates) and (ii) rate of adaptive mutation. None of these need be linearly related to the physical measures typically considered. Comparing results of experiments using different systems and comparing experimental results to models would thus be simplified by documenting the impact of the environmental conditions on key rescue parameters (Martin et al. 2013).

### Dynamics of rescue trajectories

Relatively few studies have recorded time series of demographic changes in populations exposed to stress (Perron et al. 2007, 2008; Bell and Gonzalez 2009, 2011; Samani and Bell 2010; Ramsayer et al. 2013), with only a subset linking these data to a model to quantify the rescue process. Population size trajectories can be used to quantify decay and rebound rates as well as rescue probabilities, to test whether population dynamics are density dependent, to suggest whether rescue is due to single or multiple mutations, and to indicate the repeatability of these processes across replicates. Such approaches have recently been applied to *in vitro* experiments with microbes (Martin et al. 2013; Ramsayer et al. 2013). Figure 3 illustrates examples of typical demographic trajectories leading to extinction or rescue in a bacterial population exposed to an antibiotic *in vitro*. In closer connection to the conservation field, a few studies have conducted experiments on *Drosophila* exposed to high temperature, salinity or ethanol (Frankham et al. 1999; Bijlsma et al. 2000). Although not directly dealing with evolutionary rescue, these studies take the important step of allowing extinction in experimental populations, while demonstrating the potential to expand laboratory studies beyond microbes. Future such studies could be strengthened by reporting population sizes over time, not only the endpoint of extinction.

Demographic trajectories are also sometimes available for rescue in natural settings, namely the emergence of drug resistance in treated patients. An early example studying multiple myeloma patients receiving chemotherapy fit a simple population dynamics model (one-step rescue) to estimate the decline rate of the sensitive population, the growth rate of the resistant population and the pre-existing proportion of resistant cells (Hokanson et al. 1977). A more recent example estimated parameters by fitting a viral dynamics model to viral load measurements taken in a clinical trial of a new Hepatitis C antiviral (Rong et al. 2010). These approaches could be improved by accounting for the inherent stochasticity in rescue: probabilistic model predictions can be fit to data using maximum likelihood or Bayesian methods. Nevertheless, these cases exemplify the feasibility of data collection and interpretation in clinical settings. Furthermore, data sets tracking both demographic and genetic trajectories (e.g. Sarrazin et al. 2007) are becoming increasingly available with the expansion of sequencing technologies. On the other hand, wild populations of macro-organisms present greater challenges, regarding both accurate census-taking and even identifying potential rescue situations (Gomulkiewicz and Shaw 2013). Thus, very few data sets suggesting evolutionary rescue, especially with demographic trajectories, are yet available (reviewed in Carlson et al. 2014). Nonetheless, practical steps to address empirical challenges in natural populations have recently been proposed (Gomulkiewicz and Shaw 2013).

## Areas for exchange and future work

We have illustrated that the conservation and medical communities studying rescue are quite separate. Researchers approach adaptation and extinction with different perspectives and sometimes hold divergent views on key concepts. Different motivating biological systems are surely in part responsible, but isolated historical development has also had an influence. Indeed, the basic modelling approaches used on both sides lend themselves to a more generic context than that in which they have been placed. Similarly, in experimental work, both sides rely on the same set of model systems that are amenable to study in the laboratory. Integrating conservation and medical views could yield deeper insight into several key questions that are common to both sides, although in part emphasized to different degrees. We identify the following four themes as important topics (although not a comprehensive list) requiring further development: genetic basis of rescue, standing versus *de novo* genetic variation, optimal selection strength, and spatiotemporal heterogeneity.

### Genetic basis of rescue

Determining the genetic basis of rescue – that is, the number and effect size of genetic changes – is essentially an empirical question and has been identified as a priority for experimental work (Gonzalez et al. 2013). Among natural populations of macro-organisms, there are examples of adaptation both via many small-effect changes and via few large-effect changes, arguably with an observation bias favouring the latter, but general tendencies are unclear (Hendry et al. 2011; Barrett and Hendry 2012). Drug resistance tends to be attributed to few large-effect loci, for instance mutations repeatedly seen to arise in clinical settings (Johnson et al. 2011), and it can sometimes be confirmed experimentally that a particular mutation is sufficient to confer resistance (see Empirical approaches). Generally, the rescue setting could favour adaptation via fewer, larger-effect genetic changes (and thus more parallel evolution) than in a stable population, because drift in declining populations tends to eliminate a larger set of mildly beneficial alleles (Otto and Whitlock 1997).

Rescue models have so far dealt with two extremes in genetic bases. Discrete genetic models start from a single large-effect locus; despite some progress in extensions to multistep adaptation (Ribeiro and Bonhoeffer 2000; Komarova and Wodarz 2005; Martin et al. 2013), they become increasingly cumbersome with added loci and rarely include recombination. Quantitative genetic models, in contrast, assume that many alleles cosegregate. These models typically require free recombination and/or high mutation rates, although evolutionary dynamics at intermediate recombination rates have recently been analysed (Weissman and Barton 2012) and might be transferred to rescue models. There is a need for models bridging the gap between these extremes; simulation techniques specifying finite, but possibly many, loci with specified effects (Bürger and Lynch 1995; Boulding and Hay 2001) could be useful here. Recent simulation studies have indeed relaxed the assumption of free recombination, with the initial finding that linkage can impede rescue in spatially structured, locally adapted populations (Schiffers et al. 2013; Bourne et al. 2014). Few studies have yet addressed the impact of the assumed number and effect size of loci on rescue dynamics, although one model has suggested that the chance of rescue from SGV tends to decrease as a fixed total benefit is divided among more loci (Gomulkiewicz et al. 2010). Finally, rescue models have largely neglected certain genetic features, including epistatic interactions among loci and the role of horizontal gene transfer in bringing new genetic material into a population.

Even if one or few loci are sufficient to confer drug resistance, genetic changes at other loci – such as compensatory mutations – could modify fitness and thus rescue probability. Furthermore, while beneficial mutations are usually considered in disconnect from concurrent deleterious mutations at other sites, the latter also affect the chance of survival and even become decisive in proposed therapies employing ‘lethal mutagenesis’ (Anderson et al. 2004). Note that this spectrum of mutational effects is intrinsic to quantitative genetic models via the phenotypic landscape, although in a constrained manner. Overall, models using more complex genetic bases may also be relevant in drug resistance contexts. Indeed, the medical field could adopt tools from the conservation field to investigate these effects.

### Standing versus *de novo* genetic variation

The contribution of standing versus *de novo* genetic variation is a major question in evolutionary rescue settings (Barrett and Hendry 2012) with applied implications in both fields. The conservation strategy of genetic rescue, that is, introducing genetically distinct individuals into a population at risk (Hedrick and Fredrickson 2009), is essentially a manipulation of SGV. In medicine, the extent of pre-existing resistance has implications for determining the optimal dose or combination of drugs (Ribeiro and Bonhoeffer 2000; Read et al. 2011) and timing of treatment initiation (Ribeiro and Bonhoeffer 1999).

The two communities appear to have independently reached similar conclusions: SGV is argued to make the predominant contribution to rescue over wide ranges of relevant parameter space (Bonhoeffer and Nowak 1997; Ribeiro and Bonhoeffer 2000; Komarova and Wodarz 2005; Barrett and Hendry 2012), especially when cost of mutations in the ancestral environment is low and/or the wild type decays rapidly in the new environment (Ribeiro and Bonhoeffer 2000; Martin et al. 2013; Orr and Unckless 2008, 2014). However, fitness values in different environments are typically treated as independent parameters, thus ignoring potential correlations between degree of adaptation and cost (Orr and Unckless 2008). Furthermore, conclusions to date are based on a limited set of analysed scenarios and generally lack empirical testing.

Rescue models in both fields separating the contributions of SGV and *de novo* mutation share a number of common assumptions. Firstly, they have treated only one or few large-effect loci, mainly in haploid organisms (but see Orr and Unckless 2008). Quantitative genetic or many-locus models have not made an explicit comparison of contributions, although simulations sometimes include *de novo* mutation. Nonetheless, a many-locus genetic basis could alter the chance of acquiring the necessary genetic variation, and genetic architecture and epistasis will affect maintenance of alleles (Willi et al. 2006). Secondly, models have assumed simplistic demographic history: at the time the environment changes, the population is either at mutation-selection(-drift) balance, or has grown exponentially from a small clone, with mutations arising stochastically. More complex population history (demography, selection, gene flow and population structure) could clearly affect the SGV available. Finally, models have assumed a single stepwise change in environment and often neglect density dependence. Continual environmental change or ecological feedbacks can yield ongoing temporal changes in fitness, differentiating the fixation probability of alleles from the SGV or arising *de novo* (Uecker and Hermisson 2011; Alexander and Bonhoeffer 2012).

At this point, it is unclear whether standing and *de novo* genetic variation play different roles across biological systems. A number of qualitative differences among organisms could be influential: for example, erosion of SGV through inbreeding (Bijlsma and Loeschke 2012) is only relevant in sexually reproducing species, and effects of dominance on maintenance and fixation of alleles (Barrett and Schluter 2008) only in polyploids. Furthermore, magnitudes of key rates (those of reproduction, mutation and environmental change) vary widely, resulting in dramatically different timescales on which genetic variation is generated. For example, verbal arguments emphasizing the importance of SGV often point to the delay for new mutations (Barrett and Hendry 2012), critical in a rescue situation. However, for organisms with rapid turnover and mutation (e.g. RNA viruses), the magnitude of this effect may be small (Alexander and Bonhoeffer 2012). The suggestion that *de novo* mutation gains importance under temporally gradual environmental change and/or short generation times (Barrett and Hendry 2012) also remains to be tested with models.

### Optimal selection strength

A natural applied question is how we can best manage selection pressures to promote a desired outcome in a population. The primary environmental change is under direct control in drug treatment, where the design of treatment regimes (dose, timing, combination of drugs) is a major concern. While control is less direct in conservation settings, interventions nonetheless manipulate selection pressures, for instance by altering spatial features of the habitat (e.g. designating protected areas or corridors).

Under environmental change, strong selection has two opposing effects: it induces greater demographic cost but faster adaptive response (Bürger and Lynch 1995; Bonhoeffer and Nowak 1997; Osmond and de Mazancourt 2013). Although both fields recognize this dual role, differing model structures and usage of similar terms for subtly different concepts appear to have led to divergent conclusions regarding optimal selection strength.

Quantitative genetic trait-based models deal with strength of stabilizing selection about an optimal trait value, indicated by the inverse of the width of the fitness function. A narrower fitness function exacts a larger demographic cost, and thus if adaptation is towards a fixed novel optimum, ‘stronger selection’ is always detrimental to rescue (Gomulkiewicz and Holt 1995). With a continually moving optimum, however, weak selection can make adaptation too slow to track the optimum, implying that intermediate ‘selection strength’ best promotes rescue in some parameter ranges (Bürger and Lynch 1995; Kopp and Matuszewski 2014). In any case, more severe environmental change (larger distance or rate at which the optimum moves) is disadvantageous to rescue in these models (Lynch and Lande 1993; Bürger and Lynch 1995; Gomulkiewicz and Holt 1995; Gomulkiewicz and Houle 2009; Kopp and Matuszewski 2014).

Models directly specifying fitness do not have a corresponding measure of stabilizing selection strength via the trait-to-fitness mapping. Instead, selection strength is an emergent property of specified demographic parameters in a given environment and can change over time due to ecological effects, if these are modelled. Severity of environmental change is indicated by wild-type fitness in the novel environment, which depends on dose and efficacy of drugs in medical settings. Thus, ‘strength of selection’, that is relative fitness advantage, of resistance is closely tied to severity of environmental change. Conventional medical wisdom holds that severe treatment best promotes eradication, by maximizing decline of the predominant drug-sensitive population and minimizing *de novo* mutation (Read et al. 2011). However, this idea has recently been challenged on the grounds of ‘competitive release’: when the drug-sensitive population declines, diminished competition for host resources can enhance rescue by drug-resistant strains (de Roode et al. 2004; Gatenby et al. 2009; Alexander and Bonhoeffer 2012; Huijben et al. 2013). In some cases, this effect is strong enough to imply that an intermediate treatment severity is optimal for eradication (Read et al. 2011). Although primarily highlighted in disease contexts, this effect has also been found in a more generic rescue model (Uecker and Hermisson 2011; Uecker et al. 2014).

Thus, apparently contradictory conclusions – namely that intermediate selection strength is optimal for rescue versus for eradication – arise from modelling different factors. An intermediate level of *stabilizing selection* can promote rescue in trait landscape-based models with continual environmental change. On the other hand, intermediate *relative fitness differences* between genotypes, arising directly from intermediate severity of abrupt environmental change, can promote eradication when competition significantly limits growth of resistant/viable populations, an effect that can only arise in models incorporating density dependence. Our understanding of optimal selection strength thus remains to be integrated across scenarios.

Finally, we note that selection can vary not only in intensity, but also in timing within an organism's life cycle and in ‘dimensionality’ (number of stressors; Hendry et al. 2011). Multiple simultaneous stressors are common in natural settings (e.g. alteration of a suite of climatic features; exposure to multiple drugs or pollutants), but have only infrequently been addressed by rescue models and experiments. Quantitative genetics often deals with selection on multivariate traits, and this theory has been placed in the rescue setting (Gomulkiewicz and Houle 2009). However, the fitness landscape in multidimensional trait space is still characterized by a single peak, with fitness simply determined by distance from the optimum. On the drug resistance side, several authors have modelled combination therapy (e.g. Ribeiro and Bonhoeffer 2000; Komarova and Wodarz 2005; Colijn et al. 2011), but typically characterizing total drug effects with a single parameter. A small but growing body of literature is investigating drug interactions through both models and experiments (Hegreness et al. 2008; Michel et al. 2008; Torella et al. 2010; Ankomah and Levin 2012; Ankomah et al. 2013; Pena-Miller et al. 2013), while a few models incorporate host immune response as well as drug action (Handel et al. 2009; Ankomah and Levin 2014). However, there appear to be few other studies in either field that consider environmental change via multiple stressors, particularly with nonadditive effects. Importantly, rescue theory suggests a basis for comparison, both theoretical and empirical, among multifactorial stresses, given by their net impact on key rescue parameters (see Empirical approaches).

### Spatiotemporal heterogeneity

Both nature and intensity of selection pressures can vary in space and time. Our understanding of the effects of spatiotemporal heterogeneity on rescue is still in its infancy, and cross-field exchange of emerging work could accelerate progress. A comprehensive review of the complex effects found thus far is beyond the scope of this article, but in this section, we provide pointers to references on each side to support this exchange. Moreover, a few quite general analytical frameworks have recently been developed on both sides (Foo and Michor 2010; Uecker and Hermisson 2011; Kirkpatrick and Peischl 2013) and could be further exploited in developing a unified understanding of spatiotemporal heterogeneity.

Habitats clearly show spatial structure, both for macro-organisms in the wild and pathogens within a host. Models in each field have tackled spatial structure using a variety of analytical and simulation techniques (e.g. Pease et al. 1989; Kepler and Perelson 1998; Boulding and Hay 2001; Greulich et al. 2012; Hermsen et al. 2012; Schiffers et al. 2013; Bourne et al. 2014; Uecker et al. 2014). Spatially explicit models necessarily raise the issue of migration. Gene flow can either help or hinder local adaptation (Garant et al. 2007), and additional complexities arise in the rescue setting due to the interplay of demographic and genetic effects. Spatially variable severity of environmental change implies that populations can decline slower or even grow in certain locations, which retards overall demographic decline and can create source–sink dynamics (Holt and Gomulkiewicz 2004; Uecker et al. 2014). Presence of sources, for example tissues where drugs penetrate poorly, can promote rescue (Kepler and Perelson 1998). Meanwhile, resistant/viable types face relaxed competition in locations where environmental change is more severe (Greulich et al. 2012; Hermsen et al. 2012; Uecker et al. 2014). Finally, spatial structuring affects the SGV harboured by a population and thus its contribution to rescue (Bakker et al. 2010), even if environmental change is homogeneous. Moreover, spatially heterogeneous local selection pressures can constrain global spread of rescue mutations arising in locally adapted genetic backgrounds (Schiffers et al. 2013; Bourne et al. 2014).

Temporal patterns of environmental change have typically been considered simplistically in both models and experiments, often imposed as a step change. A few theoretical studies have addressed more complex patterns, including environmental stochasticity modifying a general environmental trend (Bürger and Lynch 1995); varying rates of decay or oscillations (Wu et al. 2014); and pharmacokinetics in medicine (Lipsitch and Levin 1997; Jumbe et al. 2003). Both theoretical (Wu et al. 2014) and experimental (Lindsey et al. 2013) work so far suggests that more gradual environmental change improves chances of rescue.

Spatiotemporal variation not only in intensity, but also in the nature of the stress, arises with multifactorial environmental changes. A review of models and experiments dealing with usage of multiple drugs or pesticides on a host population scale concluded that greater heterogeneity of selection pressure appears to yield more sustainable population control (REX Consortium 2013). However, the limited number of studies addressing this high-dimensional problem calls for further investigation into the optimal strategy applying multiple stressors.

## Conclusions

Evolutionary rescue is at the heart of diverse applied problems. While conservation biologists aim for rescue and medical doctors for eradication of target populations, both communities face the same conceptual questions. Here, we have brought together literature from both sides to illustrate that insights relevant to both fields can come from diverse contexts.

Integrating evolutionary and ecological processes on a common timescale, along with bidirectional feedback between demography and genetics, presents challenges for theoreticians. These have been met using different tools in the medical and conservation communities. Sharing techniques could thus accelerate progress, particularly concerning aspects that are relevant across biological systems but so far addressed to a greater extent in one field than in the other. For example, models developed for conservation settings have dealt with a wider variety of genetic bases of adaptation (many small-effect loci or few large-effect loci), which could prove useful when studying pathogens facing complex treatment regimes. Meanwhile, ecological feedbacks are intrinsic to ‘compartmental’ (ODE-based) drug resistance models, whereas density-dependent processes have less frequently been included in other types of rescue models. Stochasticity – a crucial aspect whenever the rescue outcome is in question – has been addressed to variable degrees by different modelling approaches. While quantitative genetic and compartmental models have mainly treated stochasticity only in simulations, discrete genetic and Luria–Delbrück style models have advanced analytical treatments, at the expense of some model complexity. Extending stochastic modelling and inference frameworks to a broader range of models is thus an important common goal. Finally, the complexities of spatiotemporally heterogeneous environmental change could be more deeply understood by linking emerging work in both fields.

Basic rescue models need not be divided by the applied field in which they are grounded, and indeed most are not equipped to identify what could be distinguishing features among biological systems. As we have seen, superficially different starting points can lead to equivalent mathematical results. Linking models in a common framework clarifies their essential features and suggests extensions that may have been overlooked. We thus identify more fundamental differences between models, including their treatment of genetic basis, density-dependent demography, and stochasticity. These features do not respect the boundaries of applied fields, but rather challenge both sides to understand their consequences. Experimental testing is likewise a common ground between fields. Reporting experimental conditions and results in similar terms (e.g. quantifying stress in terms of decay rate of the initial population) would make it easier to identify general patterns across systems, while recording both demographic and genetic time series is important for comparing data and models.

We argue that a unification of rescue theory will yield not only more efficient progress, but also key insights made precisely by digging deeper into similarities and differences across biological systems. A truly comprehensive understanding of rescue necessarily includes both conservation and medical applications and is crucial for addressing challenges facing society in both contexts.
